# Is the 3D exoscope better than the surgical microscope in parotid surgery: a prospective, randomized single-center study

**DOI:** 10.1007/s00405-021-06876-5

**Published:** 2021-05-28

**Authors:** Ewelina Bartkowiak, Łukasz Łuczewski, Jadzia Tin-Tsen Chou, Małgorzata Wierzbicka

**Affiliations:** Department of Otolaryngology and Head and Neck Surgery, Poznań University of Medical Sciences, Poznan, Poland

**Keywords:** Exoscope, Microscope, Parotid gland surgery, Parotidectomy, Ergonomics

## Abstract

**Background:**

High-definition, three-dimensional (3D) exoscopes are being used to perform a growing number of head and neck surgeries. However, the use of the 3D exoscope in parotid gland surgery has not been previously described. Our initial experience with the VITOM 3D exoscope in the surgical treatment of parotid gland tumors is detailed here.

**Methods:**

We made a prospective study of patients with benign parotid gland tumors indicated for surgical resection. Between January and December 2018, patients were randomly assigned to undergo surgery assisted with the VITOM 3D system (*n* = 31) or an operating microscope (*n* = 40). Visualization quality (greater auricular nerve, digastric muscle, tragal pointer), operating time, conversion rates, and surgical outcomes were compared.

**Results:**

A total of 71 patients underwent superficial (*n* = 18) or total parotidectomy (*n* = 53). No exoscope-related complications were observed. Five patients undergoing exoscope-guided deep lobe surgery required intraoperative conversion to a microscope. No differences were observed in the subjective quality of intraoperative visualization of key anatomical structures. However, a significantly higher percentage of patients in the exoscope group developed transient facial nerve paralysis (*n* = 9; 29% vs. *n* = 4, 10%).

**Conclusions:**

These findings suggest that the VITOM 3D is a valid visualization tool for parotid gland surgery, comparable to the operating microscope but with higher resolution 3D visualization, an increased degree of freedom of movement, and better ergonomics. However, the high rate of transient nerve palsy, possibly related to decreased depth perception and the brief learning curve, merits further investigation.

## Introduction

In the last decade, the development of three-dimensional (3D) surgical visualization systems—the 3D exoscope—has given surgeons a viable—and potentially superior—alternative to conventional operating microscopes and endoscopes. Like the operating microscope (and in contrast to endoscopes), the 3D exoscope is an external device with dual image sensors for 3D visualization. In recent years, the 3D exoscope has become an established tool for neurosurgical visualization [1–5]. Although operating microscopes provide comparable illumination and magnification, visualization of the surgical field is limited to the surgeon and assistant, leaving the other members of the surgical team with only a limited view of the depth of the surgical field and important anatomical details. In contrast, images from the 3D exoscope are displayed on a large monitor, allowing excellent visualization of the surgical field for all personnel in the operating theatre, and permitting visualization of stereoscopic images in three-dimensions using 3D glasses [4-7].

The VITOM 3D exoscope (Karl Storz, Tuttlingen, Germany) is a high-definition exoscope, reported to have been first used in head and neck surgery for free flap head and neck reconstructions. Studies have shown that this device provides sufficient access, reach, and visualization to successfully perform free flap harvesting and microvascular anastomosis [8], making it a superior option to conventional operating microscopes [9]. Some authors have even reported the feasibility of using the exoscope to harvest chimeric myofascial/fasciocutaneous anterolateral thigh free flaps and to perform microvascular anastomosis [10]. To our knowledge, the 3D exoscope has not yet been evaluated in parotid gland surgery.

In this context, we conducted the present prospective, proof-of-concept randomized study to evaluate the feasibility and effectiveness of the VITOM 3D system versus a conventional operating microscope in the surgical resection of benign parotid gland tumors. We compared the two techniques to assess the differences in quality of intraoperative visualization of key anatomic structures, duration of surgery, complication rates, the incidence of permanent or transient facial palsy, and surgical conversion rates.

## Materials and methods

This was a randomized clinical trial carried out at the Department of Head and Neck Surgery at Poznań University of Medical Sciences in Poland, an academic tertiary referral center. Patients diagnosed with a benign parotid tumor were qualified for parotidectomy and randomly allocated: every first patient was operated with conventional operative microscope, while every second underwent parotid gland surgery with the high-definition VITOM 3D exoscope.

### Surgical procedure

Salivary gland surgeries have well-established classification by the European Salivary Gland Society (ESGS) [11]. It includes the glandular parenchyma level removed, designated by Roman numerals I–V, and the non-glandular structures removed. Using the facial nerve divisions as a landmark, a modification of parotid Level I and II was proposed [12].

In both groups, parotidectomy was performed as follows: (1) modified Blair or facelift incision; (2) flap creation (platysma and superficial musculoaponeurotic system layer); (3) identification of the greater auricular nerve (GAN) with preservation of the posterior branch, (4) identification of the jugular vein at the anterior border of the sternocleidomastoid muscle (SCM); (5) skeletonization of the SCM anterior body; (6) identification of the posterior belly of the digastric muscle, and (7) parotid gland excision (superficial/total).

In the anterograde technique, the facial nerve is located at the angle between the styloid and posterior belly. The pes anserinus (main trunk, cervicofacial and temporofacial branches) is first prepared with fine curved blunt-tipped scissors, before the tissues overlying the facial nerve and its branches are tunnelled and spread. The parotid tissue overlying the nerve is then divided and the tumor with a cuff of the superficial lobe is removed.

In cases where the identification of the main nerve trunk was expected to be difficult (large tumor volume or tumor location in level V), the retrograde approach is preferred. The retrograde approach is guided by the mandibular branch or the buccal branch, which may be found at predictable localizations. The buccal branch usually runs transversely with the parotid duct from the anterior border of the gland and the marginal branch at the cross point with the jugular vein.

The subjective quality of intraoperative visualization of the GAN, digastric muscle, tragal pointer, pes anserinus, and the main trunk of facial nerve and branches were assessed. The first seven patients underwent classical microscopy, but the exoscope was initiated and set in motion, constituting an alternative training view of the microscopic operation. These first seven patients allowed for the refinement of technical issues in the first three days of the study, such as the inclusion of the questionnaire: “Intraoperative quality of visualization of anatomical structures”. The surgeon (MW) filed the quality of the intraoperative visualization questionnaire in the surgery protocol. Visualization quality was subjectively categorized as good, moderate or poor. These three classifications are based on the subjective possibility of undisturbed operative continuity: “good” being that the surgeon follows the details perfectly and continues the operation; “moderate” being that the surgeon has to repeatedly improve the visual acuity of the operative field, and “poor” being that the surgeon cannot safely continue the operation on account of the blurry field, without the refinement of details and the quality she is accustomed to in a microscope.

The following surgery-related variables were evaluated and compared: duration of the procedure (minutes); bleeding (yes/no), the need for conversion from exoscope to microscope (yes/no), the need for wound revision (yes/no), and temporary/permanent facial nerve injury. Persistent nerve palsy was defined as paresis lasting more than 12 months. The basis for conversion from exoscope to microscope includes decreased depth perception in the region of the tragal pointer and stylomastoid foramen, and inadequate visualization in the event of bleeding. The bleeding required a few 2 to 3-min breaks for compression and hemostasis and was considered moderate. Bleeding was considered profuse if it impaired the operative field, i.e. the procedure required multiple breaks to tamp the tissue, coagulation was too dangerous due to the wet operating field and the risk of thermal injury to the nerve, and placement of multiple ligatures on the parenchyma was necessary.

### Statistical analysis

Statistica software (v.13) was used to perform the statistical analysis. The chi-square test with Yates’ correction (if needed) was used to compare the following variables: gender, previous surgical treatment, tumor localization, mobility, skin infiltration, nerve palsy, anatomical structure, approach, type of parotidectomy, tumor diameter, bleeding, wound revision and transient postoperative facial nerve palsy. The Student *t* test was used to compare age, duration of tumor growth, tumor size, and duration of surgery. The level of statistical significance was set at *p* < 0.05.

## Results

**Fig. 1:**
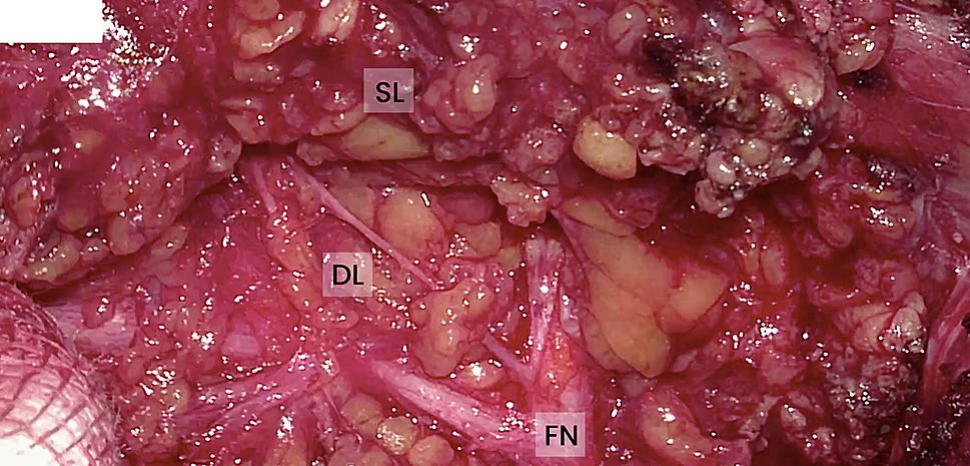
High definition 2D reproduction from exoscope of the intraoperative field, visualization of FN in the parotid parenchyma. *FN* facial nerve, *SL* superficial lobe, *DL* deep lobe

**Fig. 2:**
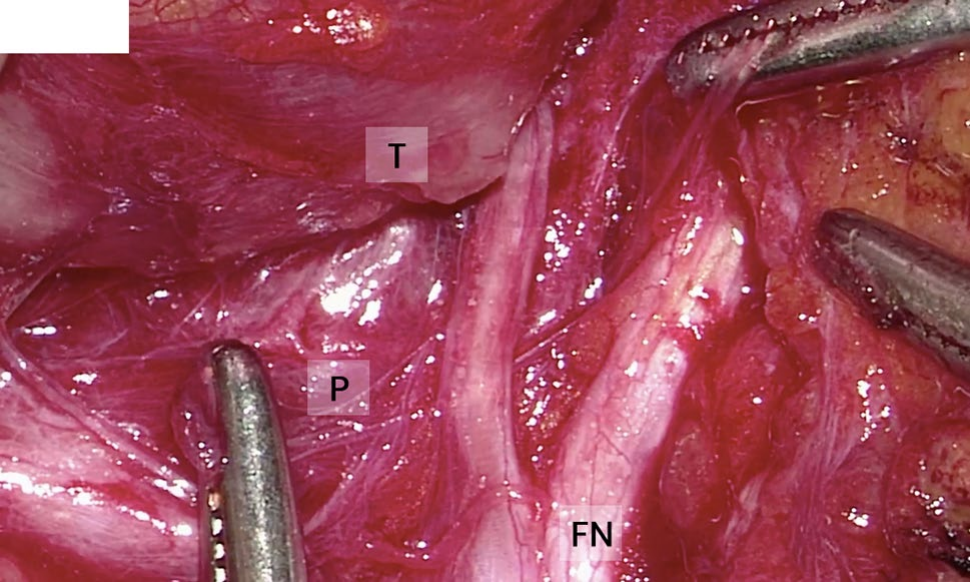
High definition 2D reproduction from exoscope of the intraoperative field, visualization of FN surrounding the tumor. *FN* facial nerve, *P* parenchyma, *T* tumor

**Fig. 3:**
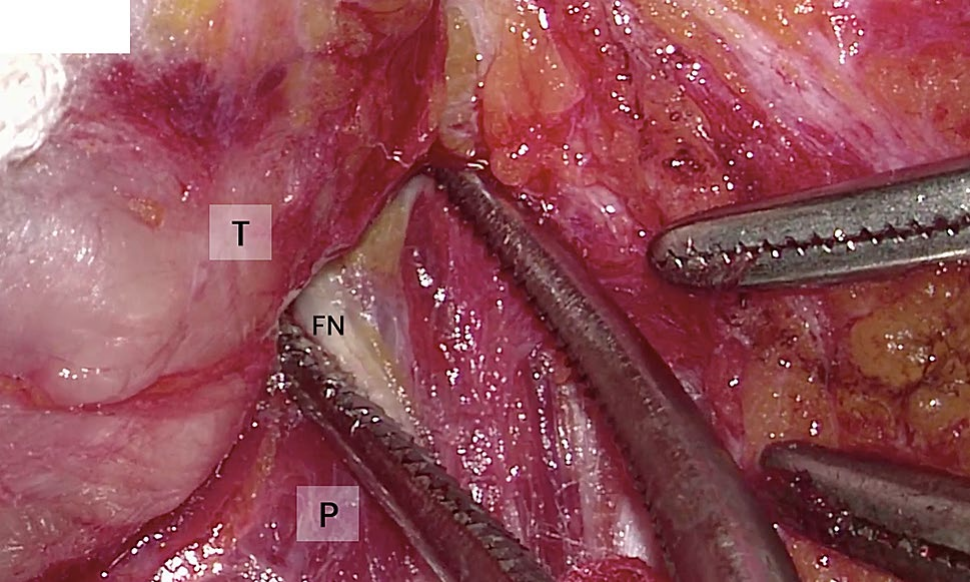
Per-operative image from microscope, visualization of FN surrounding the tumor. *FN* facial nerve, *P* parenchyma, *T* tumor

Between January and December 2018, a total of 71 parotidectomies were performed using either the VITOM 3D system (*n* = 31) (Fig. 1, 2) or an operating microscope (*n* = 40) (Fig. 3). Five patients randomly recruited into the exoscope group required intraoperative conversion and finally underwent deep lobe surgery using an operating microscope; as a result, they were further analyzed separately. Out of 71 patients, 18 underwent superficial parotidectomy (lateral: levels I, II; accessory: V) while 53 underwent total parotidectomy (levels I–V). Mean follow-up was 14 months (range: 12–18 months). Patient characteristics are shown in Table 1.

**Table 1:** Characteristics of patients (*n* = 71) who underwent parotidectomy for a benign parotid tumor

Variable	Microscope (*n* = 40)	Exoscope (*n* = 31)	*p* value
Sex, female, *n* (%)	28 (70)	12 (38.7)	0.008*
Age, years (SD)	49.2 (16.6)	56.6 (13.6)	0.044**
Previous surgical treatment, *n* (%)	5 (12,5)	3 (9,7)	0.709*
Duration of tumour growth, months (SD)	17.3 (14.5)	36.0 (68.9)	0.145**
Tumor localization, left/right	22/18	16/ 15	0.777*
Tumor mobility (good or moderate), *n* (%)	34 (85)	25 (81)	0.769*
Tumor size, cm (SD)	2.99 (1.0)	2.95 (1.1)	0.886**
Skin infiltration, *n* (%)	3 (7.5)	4 (12.9)	0.449*
Presence of preoperative facial nerve palsy (due to previous surgery or as a result of the tumor), *n* (%)	2 (5.0)	1 (3.2)	0.712*

*SD* standard deviation

*Chi–square test

**Student’s *t* test in the standard version

Intraoperative quality anatomical structure visualization: operating microscope (Fig. 3) versus the VITOM 3D exoscope (Fig. 1, 2) is shown in Table 2. There were no significant differences between the microscope and the 3D exoscope in terms of subjective measures of surgical visualization of the following anatomical structures: greater auricular nerve, digastric muscle, tragal pointer, facial nerve—main trunk, facial nerve branches. The distribution of visualization scores was uniform for both the superficial and deeper anatomical points.

**Table 2:** Intraoperative quality of visualization of anatomical structures: operating microscope versus the VITOM 3D exoscope

Anatomical structure	Microscope (* n* = 40)			Exoscope (* n* = 31)			*p* value*
	Good	Moderate	Poor	Good	Moderate	Poor	
Greater auricular nerve	32	4	4	23	4	4	0.845
Digastric muscle	19	18	3	17	9	5	0.285
Tragal pointer	20	19	1	14	13	4	0.236
Facial nerve—main trunk	19	14	7	16	10	5	0.943
Facial nerve branches	21	14	5	20	7	4	0.509

*Chi square test

Surgical variables and outcomes are shown in Table 3. Approach (anterograde/retrograde), type of parotidectomy (superficial parotidectomy for level I/II; total parotidectomy for levels I–IV), duration of surgery, potential bleeding, and transient postoperative facial nerve palsy were analyzed for both microscope and exoscope techniques. Wound revision was not necessary for either technique.

**Table 3:** Surgical variables and outcomes: operating microscope versus the VITOM 3D exoscope

Variable	Microscope (*n* = 40)	Exoscope (*n* = 31)	*p* value
Approach: anterograde/retrograde	29/11	21/10	0.663*
Duration of surgery, minutes (SD)	97.9 ( 40.8)	92.1 ( 39.8)	0.551**
Type of parotidectomy, SP/TP	10/30	8/23	0.938*
Tumor diameter > 2 cm, *n* (%)	28 (70)	22 (71)	0.929*
Bleeding, *n* (%)	4 (10)	4 (12.9)	0.701*
Wound revision, yes	0(0)	0(0)	0.906**
Transient postoperative facial nerve palsy, *n* (%)	4 (10)	9 (29)	0.039*

*SP* indicates superficial parotidectomy (level I/II), *TP* total parotidectomy (level I, II, III, IV), *SD* standard deviation, *HB* House-Brackmann classification of facial function

*Student’s *t* test in the standard version

**Chi square test

***Chi square test with Yates correction

Bolded value indicates statistical significance

In most cases (70.4% overall) the anterograde technique was used, with no significant between-group differences between the anterograde and retrograde access to the facial nerve. Approximately 70% of patients for both techniques had tumor dimensions greater than 2 cm. Both microscope and exoscope procedures had similar total operative times, at just over 90 min. The only statistically significant difference between the procedures was the incidence of postoperative transient facial nerve palsy, which was more common in the exoscope group (9/31 patients; 29%), and was associated with a more severe course. Of these 9 cases of transient nerve palsy, 7 were limited to the marginal branch and 2 involved the main trunk. The duration of transient palsy was as follows: < 12 h (*n* = 2), < 1 month (*n* = 4), and < 3 months (*n* = 3). In contrast, transient facial palsy occurred in only 4 cases (10%) in the microscope group, and all affected the marginal branch (HB1-2). Nerve palsy duration was < 12 h (*n* = 2) and < 1 month (*n* = 2).

The reasons for intraoperative conversion in 5 cases were as follows. In two patients who underwent surgery at the beginning of our learning curve (cases number 7 and 9) difficulties with intraoperative anesthetic and severe bleeding occurred. Having such a modest experience with the exoscope, the classic technique was immediately implemented. Facial palsy was not observed in these five conversion cases.

In the three other patients (cases number 16, 18 and 23) we found that the III/IV region tumors were adjacent to the facial nerve. As we had at this point noticed the slightly more frequent transient nerve palsy associated with the exoscope technique, we did not want to risk using this still new method.

## Discussion

The subjective visualization of key anatomical points, duration of surgery, complication rates, rates of permanent or transient facial palsy, and surgical conversion rates was conducted to compare the high-definition VITOM 3D exoscope to a conventional operating microscope in patients undergoing parotid gland surgery for a benign parotid tumor. Our findings show that there were no significant differences between the two techniques for most of the variables analyzed, including the duration of surgery and the subjective measures of intraoperative quality concerning the visualization of key anatomic structures (Tables 2 and 3) The only significant difference between the two techniques was the rate of transient facial nerve palsy, which affected 10% (*n* = 4) of patients in the operating microscope group versus 29% (*n* = 9) in the exoscope group.

**Fig. 4:**
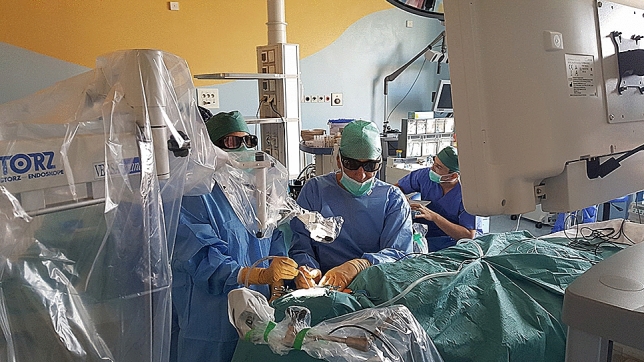
The surgical team utilizing the external monitor of the 3D exoscope for high-resolution visualization of the parotid gland

The VITOM 3D exoscope presents several important theoretical advantages over conventional instruments (operating microscopes, endoscopes, and loupes), including high-resolution 3D visualization with excellent illumination, a higher degree of freedom of movement, and excellent ergonomics with reduced fatigue [13]. In addition to these advantages, one of the main benefits of this technology is the high-definition images displayed on an external monitor, allowing the entire surgical team to observe the procedure in detail, and providing an important learning opportunity for all staff members, particularly residents in training (Fig. 4).

While several studies have described increased operative time as a limitation of the VITOM 3D system, we did not observe any difference in operative time compared to the surgical microscope. In our experience, the most important limitations of the 3D exoscope are decreased depth perception. Based on the 5 cases where it was necessary to switch to a microscope technique to excise deep-lobe tumors, we conclude that the exoscope reduces depth perception. It is very difficult to explain this finding, and it may be due to the reduced focus of light. Previous reports [14] have described similar limitations in microvascular bypass procedures. Although microvascular anastomosis is feasible under 3D exoscope visualization, depth perception at the highest magnification is inferior to that provided by a standard operating microscope [14]. For anatomical reasons, depth perception and high resolution at the highest magnification appear to be more important in the anterograde technique versus retrograde technique, although we did not observe any difference in visualization quality between the groups.

The second problem is the higher rate of transient facial nerve paresis (29% vs. 10%). The large difference between groups was surprising given the excellent visualization of the operating field and surgeon comfort. Although the reason for this difference is not clear, we suspect that it may be related to the fact that more total parotidectomies were performed using the exoscope than those performed by a microscope. Perhaps the difference is the result of a learning curve. It is also probable that the worsened sense of depth—i.e. the impaired intuitive assessment of the third dimension—resulted in more frequent and less delicate maneuvers on the facial nerve. Additionally, it should be noted that paresis of the marginal branch lasting less than 12 h should not even, *lege artis*, be classified as transient palsy. Too deep or abundant local anesthesia in the region of the mandibular angle is in fact neuropraxia, not axonotmesis.

### Study strengths and limitations

The main limitation of this study is the subjective assessment of the visualization of anatomic features in the surgical field. Another limitation is that we did not assess or compare the participating surgeons’ satisfaction with the two visualization systems, nor did we ask them to rate specific procedures with each instrument as better or worse. In contrast, the main strength of the present work is that this is, to our knowledge, the first study to describe the application of the 3D exoscope for surgical field visualization in parotid gland surgery. The prospective, randomized study design is another important strength.

## Conclusion

The findings of this study show that in patients undergoing parotid gland surgery, the VITOM 3D exoscope provides comparable results to the standard operating microscope in terms of duration of the procedure and visualization quality during surgery. Although the VITOM 3D system allows the surgeon to work more comfortably, the high rate of transient facial nerve paralysis observed in our study suggests that there is a learning curve to gain a better feel for the operating field. While the learning curve is short, it is clear that the surgeon must be highly vigilant with the facial nerves during the first several surgeries. Overall, the VITOM 3D system provides good ergonomics for the surgical staff, with high-definition images of the surgical field that is particularly useful for training residents. These findings support the use of the 3D exoscope for parotid gland surgery, although additional data would be beneficial to confirm our findings.

## Data Availability

All data generated or analyzed during this study are included in this published article (and its supplementary information files).
